# Parent and caregiver perceptions of cannabidiol products may put children at risk for unintentional exposure

**DOI:** 10.3389/fpubh.2025.1714993

**Published:** 2026-01-07

**Authors:** Michael McNally, Michael McFayden, Olivia Hime, Michael Kovasala, Grant Brown, Hunter Geneau, Simeon Holmes, Kori L. Brewer, G. Kirk Jones

**Affiliations:** 1 Brody School of Medicine at East Carolina University, Greenville, NC, United States; 2 Department of Emergency Medicine, Brody School of Medicine at East Carolina University, Greenville, NC, United States

**Keywords:** cannabidiol, CBD, exposure, Pediatrics, regulation, safety, THC

## Abstract

**Objectives:**

Rapid growth in the cannabidiol (CBD) market has been temporally associated with increasing emergency department (ED) visits related to cannabis exposure in young children. This study examined the prevalence of CBD products among individuals with and without children in the household and described their general perceptions of CBD products to assess potential risks to children.

**Methods:**

A prospective observational study was performed at a single academic hospital in the southeastern US. Flyers with a QR code linked to an online survey were posted in the adult and children’s EDs and pediatric outpatient clinic from August 2024 to May 2025. Participants indicated their age, whether they lived with/cared for children, whether they keep CBD products in the home, and answered general knowledge questions regarding CBD products. Chi-square analysis was used to compare responses across groups with *p* < 0.05 indicating significant differences.

**Results:**

Eight hundred and twenty-six eligible respondents completed the survey. Those with CBD products in their household were more likely than those without CBD products to believe these products were safe for adults (*p* = 0.03) and children (*p* < 0.001), non-addictive (*p* < 0.001), and non-fatal at high doses (*p* < 0.001). The presence or absence of children in the household did not impact overall perceptions.

**Conclusion:**

Significant inconsistency exists between households with CBD products and households without understanding the potential risks of CBD products. This may put children of households with CBD products at risk of accidental injuries. Efforts should be made towards increasing public health messaging regarding CBD products and potential THC exposure for parents, guardians, and medical professionals.

## Introduction

### Background

While both marijuana and cannabidiol (CBD) are derived from the *Cannabis sativa* or *Cannabis indica* plant, they have distinct chemical compositions and effects on the human body. Marijuana contains delta-9-tetrahydrocannabinol (delta-9-THC), the psychoactive compound that can lead to intoxication, or the “high” associated with its use. In contrast, CBD does not convert into delta-9-THC in the human body (1) and is generally considered to be non-intoxicating (2–5). In 2018, the Farm Bill was passed, which stipulated that cannabis plants and derivatives that contain no more than 0.3% THC on a dry weight basis are no longer federally controlled substances, and since this time, there has been an increase in availability, marketing, and consequently use of CBD products (6). Furthermore, despite the 0.3% THC limit stipulated by the 2018 Farm Bill, commercially available CBD products commonly contain higher levels of THC due to contamination or mislabeling; multiple studies have found THC to be present in 23–71% of CBD products, including some labeled as “THC-free” (7–9). Other CBD products have been found to include synthetic cannabinoids (10). These discrepancies pose significant risks, including the potential for intoxication, failed drug tests and serious side effects from synthetic cannabinoids (10). Additionally, the promotion and availability of these products has drastically grown in recent years (11).

### Importance

This rapid growth in the CBD market has been temporally associated with an increase in emergency department visits related to cannabis exposure in young children (age < 6 years), with a more than 1,300% increase seen between 2017–2021 (12). Data from the poison control centers around the US show that from 2017–2020, 44% of CBD-related exposures involved children under 13 years old, with 13% of cases having moderate to severe impact on the health of the patient (13). Others have reported that 36.2% of CBD-related cannabis exposures involved children from 2–12 years old and highlighted that the majority were unintentional (12).

CBD products in the form of edibles or gummies may be particularly attractive to young children who cannot differentiate them from innocuous candy or food. The widespread availability and marketing of CBD products may create a sense that the products pose no risk to children. As a result, parents may fail to secure CBD products in the same manner as they would secure prescription medications.

### Goals of this investigation

The goal of this study is to describe the prevalence of CBD products by individuals who have children in the household as well as general perceptions of CBD within this group. We also sought to determine if these perceptions differed among CBD households with and without children. The outcomes may inform future educational efforts targeted towards reducing in-home, unintentional pediatric cannabis intoxication resulting from exposure to unsecured CBD products.

## Methods

### Study design and setting

A cross-sectional, observational study was conducted over a ten-month period from August 2024 to May 2025 at a rural, academic Level I trauma center in eastern North Carolina. Adult volunteer participants were recruited through flyers posted in waiting rooms and patient areas in both the adult and children’s emergency departments, as well as the outpatient pediatric clinic. These locations were selected to increase the odds of parents and guardians of children completing the survey, which was our primary group of interest. This study was deemed exempt from review by the University and Medical Center Institutional Review Board (UMCIRB #24–000843).

### Selection of participants

**Figure 1:**
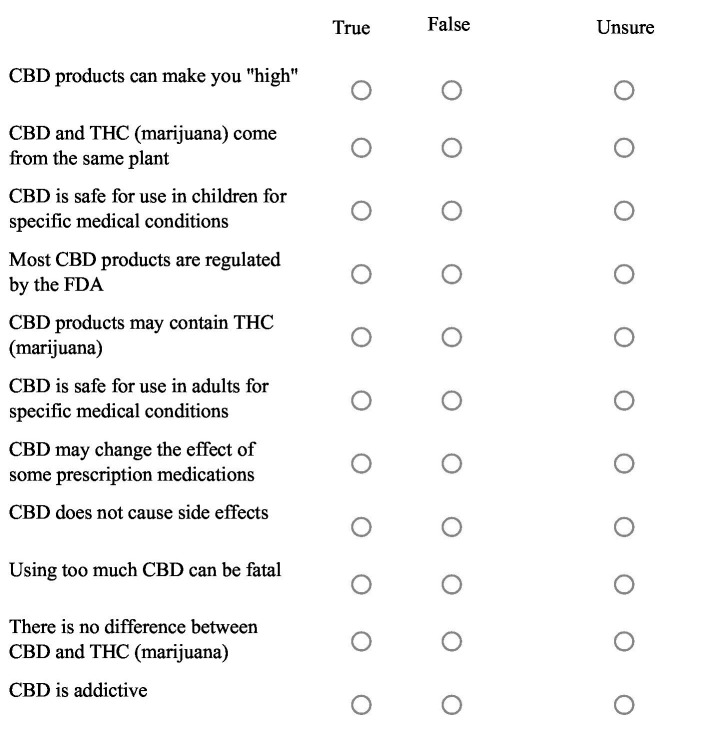
Statements used to describe general perceptions and understanding of CBD products. Respondents were instructed to choose one answer for each statement.

Each flyer featured a QR code linking to a 16-question Qualtrics survey available in both English and Spanish. Participant demographics (e.g., age, place of residence, presence of children in the home), current use of CBD products, and responses to a series of True/False/Unsure general knowledge/perception questions about CBD were recorded (Figure 1). Data was excluded if the respondent was less than 18 years of age.

### Measurements

Survey responses were collected from August 2024–May 2025 and data was analyzed using Qualtrics XM. No identifying information was collected as part of the survey, and no intervention was administered. Survey questions were constructed to reflect familiar phrasing to increase survey accessibility and engagement as this study was intended to measure general beliefs regarding CBD products (see Appendix A for full survey).

### Outcomes

The primary outcome of this study was to evaluate general perceptions of CBD products among individuals living in households with and without children, which may include parents and guardians, to determine if there is a risk to the pediatric population.

### Data analyses

Descriptive statistics were used to summarize demographic characteristics and survey responses. Percentages reported are based on the number of individuals answering each question. Chi-squared (χ^2^) analysis was performed to examine associations between categorical variables. Key comparisons included evaluating whether the presence of children in the home influenced perceptions of CBD products, with *p* < 0.05 indicating statistical significance.

## Results

Surveys were completed by 826 respondents. Limited demographic information was obtained to increase participant comfort and decrease survey length as a means to encourage completion and avoid survey fatigue. Eight hundred eight were completed in English and 11 in Spanish. Thirty surveys were excluded due to respondents being less than 18 years of age, leaving 796 for analysis. Most respondents were between 25–36 years of age (mean = 37.5 years +/− 14.3; range = 18–85). Overall, 37% of respondents (*n* = 254) reported that they or someone in their household use CBD products, with 26% of those (*n* = 60) indicating the product had been recommended by a medical provider. The most common form of CBD use was gummies (61.9%), followed by topicals (43.3%) and vapes (43.3%) (Table 1).

**Table 1:** Type of CBD products used among respondents reporting use.

CBD product type used	Percentage (%) Used
Gummies	61.9
Topicals (Oil, Lotion, Cream, etc.)	43.3
Vapes	43.3
Edibles (Brownies, Cookies, etc.)	34.2
Other	20.8
CBD isolate	10.4
Pills/capsules	8.7

When compared to respondents that do not use CBD products, those that had them in the household were more likely to believe that CBD products are considered safe for adults (37.4%, *p* = 0.030) and children (44.3%, *p* < 0.001), are not addictive (48%, *p* < 0.001), do not have any side effects (51.9%, *p* < 0.001), do not interact with prescription medications (51.2%, *p* = 0.009) and are not fatal in high doses (46.1%, *p* < 0.001).

Respondents who lived in a household with CBD products were also more likely to believe that CBD products may contain THC (41.8%, *p* = 0.012) but will not induce the ‘high’ sensation (43.1%, *p* = 0.032) (Table 2). Respondents who indicated that their CBD products were recommended by a medical provider were more likely to believe that they are safe for children with certain medical conditions (33.1%, *p* = 0.003), do not cause side effects (40%, *p* = 0.021), and that they cannot cause a “high” (32.8%, *p* = 0.029).

**Table 2:** Chi-square comparisons and percentage of respondents selecting true or false among those with CBD products in the household.

Survey statement	Percentage respondents with CBD in household responding TRUE	Percentage respondents with CBD in household responding FALSE	*p*-value
CBD is addictive	23.1	48	<0.001
Using too much CBD can be fatal	23.5	46.1	<0.001
CBD is safe for use in children for specific medical conditions	44.3	25.8	<0.001
CBD does not cause side effects	51.9	31.4	<0.001
CBD may change the effect of some prescription medications	30.4	51.2	0.009
CBD products may contain THC (marijuana)	41.8	28.6	0.012
CBD products can make you “high”	33.3	43.1	0.032
CBD is safe for use in adults for specific medical conditions	37.4	16.7	0.030
Most CBD products are regulated by the FDA*	39.7	31.5	0.104
CBD and THC (marijuana) come from the same plant*	38.3	30.3	0.145
There is no difference between CBD and THC (marijuana)*	28.3	37.7	0.262

* = relationship measured but was not significant.

For several of the survey statements, the most common response for respondents with and without CBD products in the home was “Unsure.” This included whether CBD products were FDA regulated, if they produce side effects, if they can be fatal at high doses, if they are considered addictive, and if CBD and marijuana are the same thing.

Respondents with children in the household were less likely to report using CBD (31.7% vs. 42.7% for respondents without children; *p* = 0.003). The presence or absence of children in the household did not significantly affect reported perceptions of CBD products.

## Discussion

The substantial rise in pediatric cannabis exposure over recent years is a public health concern that correlates strongly with the widespread availability and use of cannabidiol (CBD) products (13). Notably, 97.7% of these exposures occurred in residential settings, and 70% of cases resulted in severe central nervous system (CNS) depression, underscoring the risks posed by the presence of these products in households with young children (14). The proliferation of CBD products in forms such as gummies, edibles, topicals, and vape formulations has made them increasingly accessible and, consequently, more likely to be unintentionally ingested by children (14, 15).

The results of this study demonstrated significant variability in the understanding and perception of commonly used CBD products, with those respondents that use CBD being more likely to believe the products to be well-regulated and safe with no dangerous side effects. While CBD has demonstrated clinical efficacy and has received FDA approval for specific conditions like seizure disorders, most commercially available products are not FDA-regulated (16–18). These products often lack quality control and may contain THC due to contamination or mislabeling. One analysis found that THC was present in 54.55% of CBD oils, 23.81% of aqueous CBD products, and 71.43% of other CBD products (7). Another study reported that 65% of unregulated CBD products contained THC, with some products labeled as “THC-free” still containing detectable levels of THC (9). Respondents who reported using CBD in our study did indicate that they believed these products may contain THC, but also believed there was little to no risk associated with these products. This presents a clear risk to pediatric populations, particularly as THC ingestion has been associated with severe adverse outcomes, including respiratory and CNS depression (19–21). Case reports and poison control data reveal that a significant proportion of pediatric CBD exposures involve children under 13 years old, with symptoms ranging from mild lethargy and nausea to severe toxic effects requiring emergency care (14, 15, 21). In severe cases, it can result in hospitalization (19). Presumably, this is from unknown THC exposure through CBD products. For several survey items, a significant proportion of respondents were unsure whether the statements were true or false. This uncertainty may reflect confusion caused by widespread marketing amid a lack of scientific evidence definitively addressing these issues (ex: addiction, fatality, etc.).

Because the CBD market is projected to nearly double in value over the next decade (11, 22) and the perception of its safety by those that have CBD products in the home with children, our findings suggest a critical need for public education regarding product labeling, safe storage practices, and the potential harms associated with pediatric exposure. The results of our survey highlight the gaps in understanding regarding the addictive potential and psychoactive risks of these substances. Importantly, our data suggests that the presence of children in the household does not consistently correlate with safer storage or more cautious attitudes toward CBD product use.

Aside from public education, these findings support the inclusion of CBD and cannabis risk assessment in routine pediatric care. Notably, 26% of respondents having CBD products in their home indicated CBD product recommendations from their medical provider. This finding does warrant additional consideration, as it suggests some individuals may view CBD products as medically endorsed and essentially safe. As a result, these perceptions may influence storage practices, which may increase the potential risk of pediatric exposure. This result underscores the importance for medical providers to discuss not only the benefits CBD products may offer certain patients, but also the need to prioritize safe storage practices to mitigate the potential risk of pediatric exposure.

In theory, this approach would effectively resemble current screening practices, such as those for alcohol and tobacco use. Furthermore, routine screening is important when considering the potential risk for induced psychotic symptoms upon repeated THC exposure, particularly for adolescents and those with a family history of mental illness (23, 24). Educating caregivers during these routine visits may additionally reduce pediatric cannabis intoxications and prevent risk for neuropsychiatric symptoms due to exposure.

Recent evidence supports our findings, demonstrating that there has been a strongly positive correlation with the growth of CBD product availability with otherwise preventable pediatric emergency department visits, specifically cannabis related poisoning (12, 25). However, these findings are not limited to the United States, with a similar pattern reaching other countries as a result of increasingly available access to CBD products. Specifically, a recent review from Italy noted an overall increase in cannabis intoxication incidents among pediatric patients in Europe (26). Additionally, the article discussed similar regulatory concerns overseas, which have ultimately led to inconsistent consumer understanding of CBD products (26). International data continues to strengthen the conclusions from this study and reinforces the need to increase caregiver education.

### Limitations

Potential limitations included outreach due to geographical limits, lapse in regulations and variation per state, and socioeconomic status of respondents. Because the recruitment only occurred in the emergency department as well as the outpatient pediatric clinic, the sample may not serve as fully representative of the overall community. Furthermore, individuals accessing healthcare in these settings may differ from the broader community in terms of socioeconomic status, healthcare utilization patterns, and perceptions which may have otherwise influenced their survey responses. This survey took place in a rural part of North Carolina and may have yielded different responses if collection occurred in a different setting or geographical region. Respondent knowledge of terms referenced in the survey may have also altered survey responses, as survey terms were not clearly defined within the survey and relied on existing participant knowledge. No follow-up attempts were made to clarify any potential misunderstanding of survey terminology, which may have contributed to inaccurate responses and variability as well. Additionally, our survey lacked questions regarding the participant’s socioeconomic status to ensure the participant could feel comfortable answering questions, but this also limited data stratification. Future research of this nature should attempt to further stratify participants. The nature of the survey may have introduced voluntary response bias, as those who have strong positive or negative views of CBD and THC may have been more likely to complete the survey.

## Conclusion

The growth in CBD availability and use, coupled with inconsistent product quality and inadequate public awareness, may indicate a preventable cause of pediatric emergency department visits. Efforts should be made towards increasing public health messaging and education for parents, guardians, and medical providers to mitigate this potential risk of pediatric cannabis intoxication linked to CBD products.

## Data Availability

The raw data supporting the conclusions of this article will be made available by the authors, without undue reservation.

## Author’s note

Presented as patient and caregiver perceptions of CBD at NCMS LEAD in November 2024. Presented as parent and caregiver perceptions of cannabidiol products may put children at risk for unintentional exposure at ACEP scientific assembly in September 2025.
