# Supplementary material for: Parent and caregiver perceptions of cannabidiol products may put children at risk for unintentional exposure
Source: Front Public Health. 2026 Jan 7;13:1714993. doi: 10.3389/fpubh.2025.1714993 (PMC12821979; doi:10.3389/fpubh.2025.1714993)
Supplement: Supplementary file 1 [file Supplementary_file_1.docx]

Appendix A: Full survey questions with logic.

CBD Knowledge

Start of Block: Block 1

Q2 Select your language / Eleccione su idioma The goal of this survey is to learn what people know about CBD products. ***The survey will take 5-6 minutes to complete.***  By completing this survey, you are participating in a research project being conducted by Dr. Gilbert Jones, an Emergency Physician at ECU Health. Your responses are **anonymous**, and your participation is **voluntary**. ***You may choose not to answer any or all questions, and you may stop at any time.***  Please call Dr. Jones at 252-744-4757 for any research related questions or the University & Medical Center Institutional Review Board (UMCIRB) at 252-744-2914 for questions about your rights as a research participant.

Q3 What is your age?

________________________________________________________________

Q4 Do you have children that live in your home?

- No (1)
- Yes (2)

Display this question:

If Do you have children that live in your home? = Yes

Q5 How many children live in your home?

________________________________________________________________

Display this question:

If Do you have children that live in your home? = Yes

Q6 What are the ages of the children in your home (select all that apply)?

- Less than 2 years (1)
- 2-4 years (2)
- 5-7 years (3)
- 8-11 years (4)
- 12-15 years (5)
- 16-18 years (6)

End of Block: Block 1

Start of Block: Block 1

Q7 Do you or anyone in your household (including pets) use products that contain CBD?

- Yes (1)
- No (2)
- Not Sure (3)
- Prefer not to answer (4)

Display this question:

If Do you or anyone in your household (including pets) use products that contain CBD? = Yes

Q8 What type of CBD products are used in your household (select all that apply)?

- Topical (oil, lotion, cream, etc.) (1)
- Gummies (2)
- Vapes (3)
- Edibles (brownies, cookies, etc.) (4)
- Pills/Capsules (5)
- CBD Isolate (6)
- Other (7)

Display this question:

If Do you or anyone in your household (including pets) use products that contain CBD? = Yes

Q9 Has a medical provider recommended that you use CBD products for yourself or someone in your household?

- Yes (1)
- No (2)

Display this question:

If Do you or anyone in your household (including pets) use products that contain CBD? = Yes

Q10 Are the CBD products used in your household approved by the FDA (Federal Drug Administration)?

- Yes (1)
- No (2)
- Not Sure (3)

Display this question:

If Do you or anyone in your household (including pets) use products that contain CBD? = Yes

Q12 Do you or anyone in your household take medications for the following conditions (check all that apply)?

- High blood pressure (1)
- High cholesterol (2)
- Diabetes (3)
- Heart problems (4)
- Depression/anxiety/mental illness (5)
- Seizures (6)
- Blood clots (7)
- Immune conditions (ex: HIV, multiple sclerosis, lupus, psoriasis, etc.) (8)
- Erectile dysfunction (9)

Display this question:

If Do you or anyone in your household (including pets) use products that contain CBD? = Yes

Q13 Do you or anyone in your household take medications for the following conditions (check all that apply)?

- High blood pressure (1)
- High cholesterol (2)
- Diabetes (3)
- Heart problems (4)
- Depression/anxiety/mental illness (5)
- Seizures (6)
- Blood clots (7)
- Immune conditions (ex: HIV, multiple sclerosis, lupus, psoriasis, etc.) (8)
- Erectile dysfunction (9)

Display this question:

If Do you or anyone in your household (including pets) use products that contain CBD? = Yes

Q14 Do you or anyone in your household take medications for the following conditions (check all that apply)?

- High blood pressure (1)
- High cholesterol (2)
- Diabetes (3)
- Heart problems (4)
- Depression/anxiety/mental illness (5)
- Seizures (6)
- Blood clots (7)
- Immune conditions (ex: HIV, multiple sclerosis, lupus, psoriasis, etc.) (8)
- Erectile dysfunction (9)

End of Block: Block 1

Start of Block: Block 2

Q11 Indicate whether you think each of the statements below is True or False

|  | True (1) | False (2) | Unsure (3) |
| --- | --- | --- | --- |
| CBD products can make you "high" (1) |  |  |  |
| CBD and THC (marijuana) come from the same plant (2) |  |  |  |
| CBD is safe for use in children for specific medical conditions (3) |  |  |  |
| Most CBD products are regulated by the FDA (4) |  |  |  |
| CBD products may contain THC (marijuana) (5) |  |  |  |
| CBD is safe for use in adults for specific medical conditions (6) |  |  |  |
| CBD may change the effect of some prescription medications (7) |  |  |  |
| CBD does not cause side effects (8) |  |  |  |
| Using too much CBD can be fatal (9) |  |  |  |
| There is no difference between CBD and THC (marijuana) (10) |  |  |  |
| CBD is addictive (11) |  |  |  |

End of Block: Block 2

Start of Block: Block 3

Q12 Please leave any comments below (optional), and click arrow to submit.

________________________________________________________________

________________________________________________________________

________________________________________________________________

________________________________________________________________

________________________________________________________________

End of Block: Block 3
